# Combinational antitumor strategies of exosomes as drug carriers: Mini review

**DOI:** 10.3389/fphar.2022.1107329

**Published:** 2023-01-20

**Authors:** Guixiu Xiao, Zihan Xu, Feng Luo

**Affiliations:** ^1^ Lung Cancer Center, Cancer Center and State Key Laboratory of Biotherapy, West China Hospital of Sichuan University, Chengdu, Sichuan, China; ^2^ Laboratory of Integrative Medicine, Clinical Research Center for Breast, State Key Laboratory of Biotherapy, West China Hospital, Sichuan University and Collaborative Innovation Center, Chengdu, Sichuan, China

**Keywords:** exosome, engineered exosomes, drug carriers, cancer, combinational therapy

## Abstract

Cancer therapies have made tremendous progress in the last decade, but monotherapy still has apparent limitations and lacks therapeutic efficacy. Thus, the simultaneous administration of multiple drugs has been widely explored and has shown better outcomes. Exosomes, deriving from almost all living cells, are natural nanocarriers designed to deliver drugs to tumor sites. Therefore, combinational antitumor therapies based on exosomes, such as engineered exosomes and different combinations of chemotherapeutic agents, therapeutic nucleic acids, photosensitizers, immunotherapy and phytochemicals, have considerable prospects and potential for clinical translation. Here, we summarize current strategies of cancer combination therapy in exosomes and propose opportunities and challenges in the future.

## 1 Introduction

Although antitumor therapies have advanced rapidly, cancer remains one of the leading causes of mortality (Sung et al., 2021). With the discovery of non-coding RNAs (ncRNAs) and programmed death receptor 1/programmed death receptor ligand 1 (PD-1/PD-L1), as well as research on their mechanisms, the therapeutic targets of ncRNAs and immunotherapy have started a new chapter of cancer therapy (Bagchi et al., 2021; Kara et al., 2022). However, the degradation of nucleic acids *in vivo* and the limited benefits of immune checkpoint inhibitors have become new challenges (Dammes and Peer, 2020; Winkle et al., 2021; Yi et al., 2022). In addition, insufficient efficacy, side effects, rapid clearance, and resistance have limited the long-term treatments of conventional chemotherapeutic agents (Gao et al., 2022). The combination of different therapies emerges as the time required and shows considerable promise.

Exosomes (EXO), a subtype of extracellular vesicles (EVs), are generated from various cells and can be detected in body fluids. They contain proteins, lipids, and ncRNAs (miRNAs, lncRNAs, and circRNAs), which determine their properties and possess vital biological or pathologic functions in intercellular communication (Kalluri and LeBleu, 2020; Paskeh et al., 2022). Their low immunogenicity, high plasticity, biocompatibility, and potential for targeted delivery make it a promising carrier in cancer therapy (Kooijmans et al., 2021). Thus, the double-membraned vesicles started to be used to protect and deliver cargos. They successively took therapeutic agents to tumor tissue and then suppressed tumor proliferation (Thakur et al., 2022).

However, rapid clearance of some exosomes in the blood and multidrug resistance of tumors limit the efficacy of exosome-based drug delivery (Huang et al., 2017; Zhang et al., 2019). To further exert the carrier, combinational antitumor strategies of exosomes have been exploited. They have gradually shifted drug-loaded exosomes from single to combinational therapy to cope with heterogeneous cancer cells through synergistic effects. This review summarizes recent advances in exosomes as carriers for combined cancer therapy, including the methods of engineering modification and different combinations of chemotherapeutic agents, therapeutic nucleic acids (TNAs), photosensitizers, immunotherapy and phytochemicals. We also outline the challenges and new strategies, which will provide guidance for future research on multifunctional exosomes.

## 2 Engineering modification of exosomes

**FIGURE 1:**
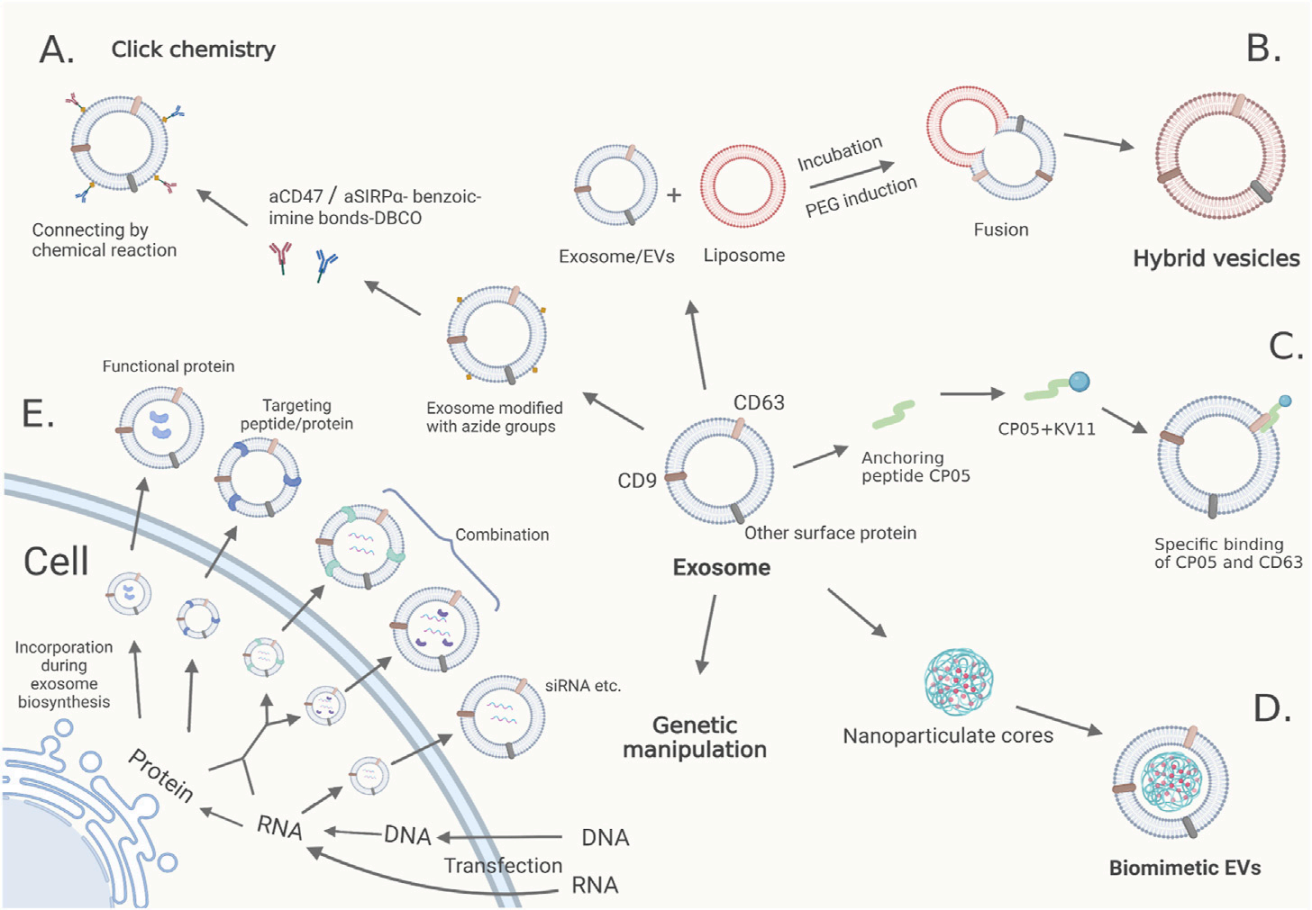
The modification methods of engineered exosomes: **(A)** Click chemistry. Exosomes are modified with azide groups through the intrinsic biosynthesis and metabolic incorporation of phospholipids. Then azide-modified exosomes are conjugated with the dibenzocyclooctynes (DBCO)-modified anti-CD47 antibody (aCD47) and anti-signal regulatory protein alpha (SIRPα) antibody (aSIRPα) linked with pH-sensitive benzoic-imine bonds. **(B)** Hybrid vesicles. Polyethylene glycol (PEG) and incubation induce efficient fusion between exosomes/EVs and liposomes. **(C)** Anchoring peptides. Anchoring peptide CP05 linked with KV11 (an anti-angiogenic peptide) bounds specifically to CD63. **(D)** Biomimetic EVs. Nanoparticulate cores are embedded into the exosomes by electroporation. **(E)** Genetic manipulation. Transfected nucleic acids or their protein producs are loaded through the intrinsic biosynthesis of exosomes.

Exosomes derived from cells are capable of targeting recipient cells through the binding of proteins with ligands and present compatibility to parental cells (Xia et al., 2020). For example, dendritic cell (DC) cell-derived exosomes express ICAM-1, which increases exosome uptake by binding to LFA-1 on the surface of activated T cells (Nolte-'t Hoen et al., 2009). However, low drug delivery efficiency, insufficient infiltration and weakened targeting capability *in vivo* require multiple modifications to solve these problems (Chen et al., 2022). These deficiencies engineering modification of exosomes endows them with not only superior targeted capacity but also longer circulation time and more muscular infiltration (Kooijmans et al., 2016). Here, we present classic methods and recent advances in the engineering modification of exosomes, including direct and indirect ways, such as click chemistry, anchoring peptides, hybrid vesicles, biomimetic EVs, and genetic manipulation (Figure 1; Supplementary Table S1).

### 2.1 Click chemistry

Click chemistry refers to rapid, spontaneous and highly efficient reactions between two chemical groups allowing for the incorporation of chemical groups with highly selective reactivity into small molecules or protein modifications without perturbing their biological function and with no membrane damage or undesirable side reactions (Peplow, 2019; Parker and Pratt, 2020). Click chemistry has become an essential tool for constructing biomolecular systems (Goyard et al., 2022). Copper-catalyzed azide alkyne cycloaddition, one of click chemistry, is a reaction between an alkyne and an azide that forms a triazole linkage (Goyard et al., 2022). Functional ligands c(RGDyK), the cyclo (Arg-Gly-Asp-D-Tyr-Lys) peptide, are conjugated onto exosomal surfaces through bio-orthogonal copper-free azide alkyne cyclo-addition and show high affinity to integrin αvβ3 in a mouse model (Tian et al., 2018). Tumor cells express CD47, which can hinder recognition by macrophages *via* the CD47-signal regulatory protein α (SIRPα) signaling pathway (Liu et al., 2015). Dibenzocyclooctyne (DBCO)-modified antibodies against CD47 and SIRPα are conjugated with exosomes modified with azide groups through pH-sensitive benzoic-imine bonds and released from exosomes in an acidic microenvironment as blocking agents (Nie et al., 2020) (Figure 1A). In addition, click chemistry has also been used in exosome tracing and synchronous imaging monitoring (Xu et al., 2020; Qian et al., 2022). However, applications of click chemistry should be accompanied by intelligent strategies because specific chemical structures are needed as prerequisites.

### 2.2 Hybrid vesicles

Hybrid vesicles are obtained by fusing exosomes/EVs with liposomes, thereby they have the advantages of both (Figure 1B). On the one hand, polyethylene glycol may trigger the fusion of EVs with functionalized liposomes to create novel biosynthetic hybrid vectors, which provides an effective way to camouflage liposomes (Piffoux et al., 2018). On the other hand, hybrid nanoparticles could load large molecules or exogenous cargo much more easily than EVs, and encapsulated genes can be expressed after endocytosis (Lin et al., 2018; Evers et al., 2022). In fact, with a deep understanding of exosome properties, it will be a pivotal method for us to adjust the proportion of different components in hybrid vesicles to produce superior materials. In addition, new techniques, such as charge-mediated hybrid fusion, open more possibilities of applying hybrid vesicles (Marusic et al., 2022).

### 2.3 Anchoring peptides

Anchoring peptides can be used as versatile targeting tools with the capacity to bind membrane molecules and antibodies. Through fusion with glycosylphosphatidylinositol anchors, anti-epidermal growth factor receptor (EGFR) nanobodies were strongly enriched in EVs and greatly improved the capacity of targeting tumor cells (Kooijmans et al., 2016). Later, the anchoring peptide CP05 was proven to have the ability to target CD63 on the exosome surface, and some researchers have tried to use CP05-modified exosomes as delivery carriers (Gao et al., 2018). For example, exosomes loaded with KV11 (an anti-angiogenic peptide) through CP05 exhibit better delivery efficiency and suppress neovascularization in retinopathies (Dong et al., 2021) (Figure 1C). Similarly, CP05 was applied to modify 3D-printed scaffolds to improve the grafting efficiency of the engineered exosomes (Zha et al., 2021). Despite promising results in other disease models, CP05 has not been explored in targeting tumor cells, which might be a research direction in the future.

### 2.4 Biomimetic EVs

Biomimetic EVs are combinations of synthetic nanoparticulate cores and biologically derived membrane coatings, which exhibit excellent pharmacokinetics and biocompatibility (Dehaini et al., 2017) (Figure 1D). For instance, EXO-PMA/Au-BSA@Ce6 nanovehicles, generated from freshly urinary exosomes, are loaded with multifunctionalized PMA/Au-BSA@Ce6 nanoparticles *via* an instant electroporation strategy, which can bypass the host immune response, prolong circulation time and delay the endocytosis of macrophages (Pan et al., 2020). In the same way, the boron nitride nanotubes loaded with doxorubicin and coated with cell membranes extracted from glioblastoma multiforme cells are enabled to penetrate into glioma tissues and greatly promote cellular internalization and antiproliferation ability as well as extending circulation time (De Pasquale et al., 2020). Given the large number of exosomes needed, taking the membrane structure from other sources into consideration seems feasible.

### 2.5 Genetic manipulation

Genetic manipulation is an indirect method of modification that requires transfection tools to deliver cargo to parent cells, increase the expression of RNAs or proteins, and finally generate exosomes with ideal targeted molecules or RNAs through a natural biogenesis process (Zhang et al., 2020; Zhang et al., 2021) (Figure 1E). Exosomes expressing cardiac-targeting peptide Lamp2b on the exosomal membrane have been obtained in this way and have been developed as a therapeutic tool for heart disease (Tian et al., 2018). Moreover, engineered EVs as versatile ribonucleoprotein (RNP) delivery vehicles for CRISPR (clustered regularly interspaced short palindromic repeats) genome editing has been explored and created a system capable of delivering RNP targeting multiple loci for multiplex genome editing (Yao et al., 2021). Furthermore, properly combining both modified donor cells and exosomes is a potential choice (Lv et al., 2020). However, it brings worries about introduced ligands interrupting the normal function of other proteins or showing up in undesirable places.

### 2.6 Other methods

Due to the negatively charged surface, cations can modify EVs by electrostatic interactions, but some cationic materials are cytotoxic and induce lysosome degradation. In addition, metabolic engineering refers to changing parental cells by using a growth medium with specific substances to promote cellular biosynthesis (Zhang et al., 2022). Research on these methods is relatively limited at present.

## 3 Combinational antitumor strategies

**FIGURE 2:**
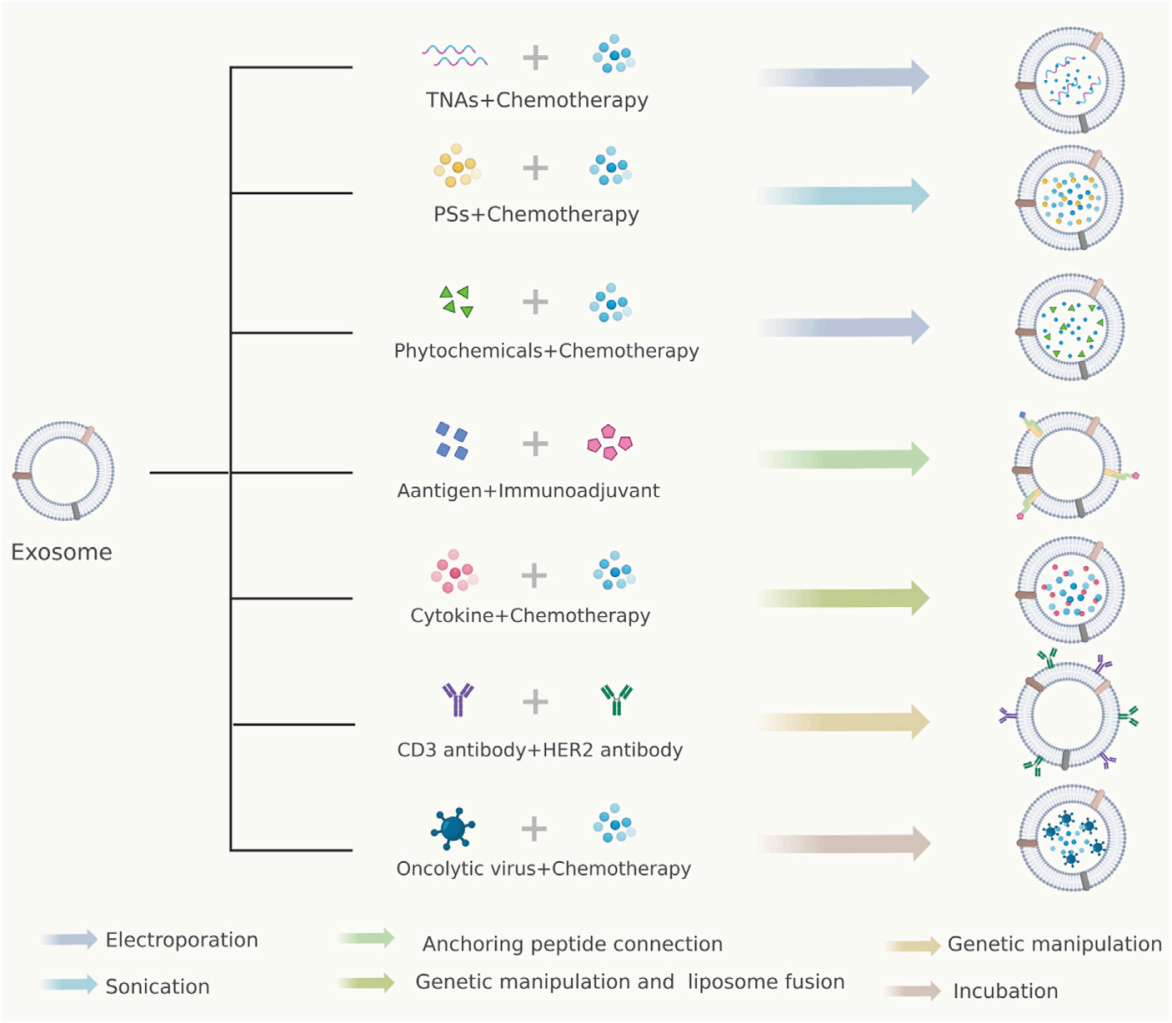
Combinational antitumor strategies of exosomes as drug carriers. Arrows with different colors represent various drug loading methods. TNAs, therapeutic nucleic acids; PSs, photosensitizers; Her2, human epidermal growth factor receptor 2.

When monotherapy is not enough to suppress the proliferation of cancer cells or drug resistance appears, combination therapy shows more efficacy. However, few drugs could reach the tumor site after systemic administration. To achieve a high concentration of drugs at the local site, nanocarriers are modified to aggregate drugs in the tumor environment. Codelivery nanocarriers have become a newly emerging strategy to magnify synergistic effects and minimize adverse reactions (Al Bostami et al., 2022). We summarize current combinational antitumor plans based on exosomes and propose some views about future challenges and development (Figure 2; Supplementary Table S2).

### 3.1 TNAs combined with chemotherapeutic agents

Many efforts have been made to illustrate the role of ncRNAs in cancer (Slack and Chinnaiyan, 2019). Numerous studies have identified specific miRNA or lncRNA participating in the development of tumor proliferation, metastasis and resistance (Wang et al., 2019). Thus, codelivery of TNAs and classical chemotherapeutic agents by exosomes is growing into an emerging plan. A recent study designed target-specific exosomes to deliver miR-21 inhibitors (miR-21i) and 5-fluorouracil (5-FU) into 5-FU-resistant HCT-116 cells, a colorectal cancer cell line with highly expressed miR-21 (Liang et al., 2020). They fused Her2 (human epidermal growth factor receptor 2) with LAMP2 (lysosome-associated membrane proteins 2), and the fusion protein expressed on the surface of exosomes facilitated cellular uptake. 5-FU and miR-21i were packaged into exosomes through electroporation by mixing an appropriate concentration. Then, the engineered exosomes were proven safe and effective both *in vivo* and *in vitro*. Another study utilized A15-EXOs to deliver cholesterol-modified miRNA (miR-159) and doxorubicin to triple-negative breast cancer (TNBC), which exhibits potent antitumor activity and precisely targeted delivery (Gong et al., 2019). In addition, a recent report identified lncRNA PGM5 antisense RNA 1 (PGM5-AS1) as a prognostic indicator correlated with better OS in colorectal cancer patients treated with oxaliplatin, and exosomes loaded with PGM5-AS1 and oxaliplatin indeed inhibited the proliferation, metastasis, and acquired resistance to oxaliplatin of colon cancer cells (Hui et al., 2022).

Engineered exosomes indeed increase the concentration of therapeutic agents in local tumors. Exosomes loaded with TNAs and chemotherapeutic drugs make it feasible for clinical translation. However, electroporation widely used in loading drugs can induce the aggregation of nucleic acids, which might cause unknown effects (Jeyaram et al., 2020). In addition, the efficiency of this approach remains to be improved. An appropriate proportion of every agent, and a suitable electroporation solution system or parameters will be one of the future research directions (Liang et al., 2020).

### 3.2 Chemotherapy-photodynamic therapy

Photodynamic therapy (PDT) is a cancer treatment that excites photosensitizers in tumor tissues by specific wavelengths of light, resulting in the generation of reactive oxygen species (ROS) and leading to cell death (Choi et al., 2022). A new combination strategy of exosomes appeared recently, which armed exosome with CD47 and ferroptosis inducer erastin (ER) on the surface together with the core of the photosensitizer Bengal (RB) (Du et al., 2021). Upon irradiation with the laser, ER and RB synergistically induced cell death in the tumor region without apparent toxicity. Cellular glutathione (GSH) can be oxidized and subsequently reduced to participate in antioxidant activity, restricting PDT, which relies on ROS. Moreover, glutamine is an essential source of nutrition for tumor cells and GSH synthesis (Altman et al., 2016). Therefore, glutamine metabolism inhibitors and PDT could be used simultaneously. Zhu et al. designed exosomes carrying aggregation-induced emission luminogens (AIEgens) and proton pump inhibitors (PPIs) to combine enhanced type-1 photodynamic therapy with tumor glutamine starvation therapy, which showed a significant antitumor effect (Zhu et al., 2022). Apart from these, the superiority of exosomes combined with PDT in real-time fluorescence imaging is visible (Pan et al., 2020; Qian et al., 2022).

Capsulated in membrane structures, such as exosomes and cellular membranes, provide the second chance to use drugs with low water solubility, adverse pharmacokinetics or biodistribution. Meanwhile, it puts forward the idea of adding antimetabolites to combined regimens based on exosomes. Studies of other agents acting inside tumor cells will unceasingly process to find more strategies for cancer therapy.

### 3.3 Immunotherapy in combinational therapy

Engineered exosomes are widely used in antitumor immunity. Bearing abundant signature proteins from their parental cells, DC-derived exosomes (DEXs) have been used as new vaccines (Naseri et al., 2020). Zuo et al. equipped DEX with a hepatocellular carcinoma (HCC)-targeting peptide-P47, an immunoadjuvant high mobility group nucleosome-binding protein 1 (HMGN1) and an α-fetoprotein epitope (Zuo et al., 2022). This vaccine system could recruit and activate DCs in tumor sites, triggering antigen-specific immune responses and inducing tumor-killing effects in orthotopic HCC mice. In the same way, α-lactalbumin (α-LA)-modified exosomes delivered the immunogenic cell death inducers human neutrophil elastase (ELANE) and Hiltonol (TLR3 agonist) to TNBC and resulted in enhanced CD8 T-cell responses in a mouse xenograft model and patient-derived tumor organoids (Huang et al., 2022). In addition, exosomes painted with a dual antibody targeting CD3 T cells and breast cancer-associated HER2 receptors can recruit not only T cells but also induce specific killing of HER2-positive breast cancer cells (Huang et al., 2022). Lung cancer cell-derived EVs could bring oncolytic viruses (OVs) and chemotherapy drugs such as paclitaxel to tumor tissue, leading to enhanced antitumor effects in nude mice (Shi et al., 2020). Exosomes as delivery carriers provides a new method to augment the effects of immunizing agents and turn cold tumors hot.

In addition, exosome delivery of small interfering RNAs (siRNAs) targeting key immune-related genes is another successful exploration of antitumor immunity. Immune checkpoint blockade induces responses well in some cancer types, whereas it presents dismal effects in glioblastoma (GBM) because of the blood‒brain barrier (BBB) and suppressive microenvironment in GBM (Lai et al., 2022). C(RGDyK)-conjugated EVs smoothly bring PD-L1 siRNA to GBM and improve the immunosuppressive microenvironment after short-burst radiation, which provides a scheme for immune checkpoint therapy in brain tumors (Tian et al., 2022). Similarly, codelivery of oxaliplatin and siRNA against galectin-9 triggers ICD and reverses immunosuppression caused by M2 tumor-associated macrophages in pancreatic ductal adenocarcinoma (Zhou et al., 2021).

Given their targeting and penetrating ability, exosomes also demonstrate powerful advantages in metastatic cancer. Lv et al. fused CD47-expressing exosomes with thermosensitive liposomes to develop a nano delivery system loaded with granulocyte-macrophage colony-stimulating factor (GM-CSF) and docetaxel, which could promote the repolarization of macrophages toward M1 and show more significant therapeutic benefits than either agent alone in metastatic peritoneal carcinoma (Lv et al., 2020; Shi et al., 2020; Garofalo et al., 2019).

### 3.4 Phytochemicals in combinational therapy

The antitumor effects of phytochemicals have been confirmed (Kola et al., 2022). Due to their low toxicity and multiple modulation pathways, phytochemicals have become a new option for cancer therapy (Gao et al., 2022). Curcumin, a plant polyphenolic compound, can suppress tumor growth through various mechanisms (Bhattacharyya et al., 2007; Sanidad et al., 2019). However, like most drugs, it cannot cross the BBB and lacks tumor-targeting ability. RGE-modified exosomes loaded with superparamagnetic iron oxide nanoparticles (SPIONs) and curcumin could cross the BBB smoothly, target glioma accurately, and markedly improve the antitumor effects (Jia et al., 2018). Phytochemical-based combinational regimens are limited by the volume of exosomes for multiple drugs, but hybrid vesicles or a combination of other therapies could provide potential solutions.

## 4 Discussion

Currently, the trend of combinational therapy is overwhelming because of poor therapeutic effects of single agent and drug resistance. Although several nanomaterials have been applied in codelivery, cytotoxicity and low permeability restrict their application (Chen et al., 2021).Exosomes as delivery carriers possess many advantages, such as high efficacy, low toxicity, low immunogenicity, low off-target risks, bioavailability and stability. Delivery by exosomes increases the efficiency of cancer treatments and reduces unwanted drug aggregation. Exosomes can penetrate biotic barriers and deep tissues to target tumors, which endows them with significant advantages in cancer therapy, especially in metastatic carcinoma. Despite the great potential of exosome-based therapies, unscalable production of exosomes remains a challenge to translate these therapies to clinical practice. First, a standard protocol and commercial production of exosomes are needed. Given that large amounts of exosomes are needed, numerous technologies are under study to increase production (Debbi et al., 2022; Seo et al., 2022). Some alternative sources are also being explored, such as exosomes extracted from milk and plants (Badawy et al., 2018; Dad et al., 2021; El-Kattawy et al., 2021; Zhong et al., 2021). The digestive tract can absorb them, but the concentration of the contents reaching the treatment site is another question to consider. Second, how to optimize loading efficiency has always been a problem. Since passive diffusion is the primary mechanism of the present methods, such as incubation, freeze—thaw cycling, sonication, and electroporation, the loading efficiency is unsatisfactory. The interesting idea that the self-assembly of exogenous siRNAs could be encapsulated into secretory exosomes in the liver and delivered to the targeted tissue attracted our attention (Fu et al., 2021). Although it is an innovation of active loading, whether it can be artificially controlled and improve the loading efficiency needs more evidence. Finally, more strategies remain to be developed to provide more superior choices. For example, the delivery of CRISPR—Cas by exosome has made some progress, and if self-assembly of CRISPR *in vivo* could be realized, it will becomes a powerful tool for gene therapy (Wan et al., 2022).

Engineered exosomes have been developed for dual/multiple drug codelivery, which can improve pharmacokinetic behavior and tumor accumulation capacity as well as achieve tumor site-targeted delivery. Drugs based on exosomes, like zofin for COVID-19, have been approved by FDA for clinical trials, but their applications in cancer focus on diagnosis or safty assessment now (Mitrani et al., 2021). There is no doubt that exosomes transform conventional disease treatment into a new era of cell-free therapy and are promising for realizing clinical translation.

## 5 Conclusion and perspectives

This review has summarized the recent advances in exosomes as carriers for combinational antitumor therapies, including the methods of engineering modification and different combinations of chemotherapeutic agents, therapeutic nucleic acids, photosensitizers, immunotherapy and phytochemicals. Despite the progressive advances, there is still a gap between experimental research and clinical application. Further studies are quite essential to explore the properties of various exosomes for further application. In addition, it is necessary to further clarify how to select appropriate loading methods and route of administration to promote loading efficiency and absorption of cancer cells for different target tissue and drug’s chemical composition. Exosomes play an important role as carriers in combination antitumor therapy, but the choice of combination therapy strategy is critical because not all combinations enhance antitumor effects and combination strategies might increase the risk of adverse reactions. For clinical application, completely pure exosomes, without any biological/biochemical contaminations, have not been obtained, so how to further purify the acquired exosomes is an important step in achieving clinical transformation. Although there are many challenges in the emerging field, multifunctional exosomes have shown significant effects in a variety of cancer animal models, suggesting great potential for future clinical transformation.
