# Supplementary material for: Combinational antitumor strategies of exosomes as drug carriers: Mini review
Source: Front Pharmacol. 2023 Jan 20;13:1107329. doi: 10.3389/fphar.2022.1107329 (PMC9897293; doi:10.3389/fphar.2022.1107329)
Supplement: Supplementary file 1 [file Table1.DOCX]

**For the convenience of the reader, we use a different reference style here.**

**Supplementary Table 1.** The modification methods of engineered exosomes.

| **Modification methods of exosome** | | **Exosomes source** | **Model** | **Outcome** | **References** |
| --- | --- | --- | --- | --- | --- |
| **Click chemistry** | c(RGDyK) peptide has been conjugated to the exosome surface by bio-orthogonal copper-free azide alkyne cyclo-addition | mesenchymal stromal cell | transient middle cerebral artery occlusion mice model | improve the capacity of targeting lesion region of the ischemic brain | [1] |
|  | azide-modified exosomes are conjugated with the dibenzocyclooctynes -modified anti-CD47 antibody and anti- SIRPα antibody linked with pH-sensitive benzoic-imine bonds | M1 macrophage | tumor-bearing mice of breast cancer (4T1) | target tumors through the anti-CD47 antibody, block SIRPα and CD47 and abolish “don't eat me” signaling and improve phagocytosis of macrophages | [2] |
|  | azide-functionalised AlexaFluor®488 (AF488) dye is labelled by copper-free click reaction | B16-F10, PANC-1 and HEK-293 cells | NSG mice bearing subcutaneous human pancreatic cancer (PANC-1) or mouse melanoma (B16-F10) | enable intracellular tracking and uptake quantification | [3] |
| **Hybrid vesicles** | PEG induce the fusion between EVs of different cellular origins and liposomes | HUVEC, murine MSC and MDCK | - | hybrid vesicles deliver mTHPC in cancer cells more efficiently than liposomes | [4] |
|  | incubate the original exosomes with liposomes | HEK293FT Cell | - | hybrid nanoparticles efficiently encapsulate large plasmids and can deliver CRISPR–Cas9 system in MSCs | [5] |
|  | cell membrane fused with synthetic phospholipids by using lipid-film hydration and extrusion | Prostate cancer cells (U937) | xenograft-bearing mice of prostate cancer | dually targeted PSA/PSMA-Hybrid significant tumour growth delay in vivo | [6] |
|  | EVs and liposomes are merged via thin-film hydration and extrusion | SKOV3 and cardiac progenitor cells | - | EV–liposome hybrid nanoparticles can be loaded with siRNA and retain functional properties of EVs | [7] |
| **Anchoring peptides** | GPI-linked EGFR nanobodies were displayed on EV surfaces | mouse neuroblastoma Neuro2a cells | - | improved EV binding to tumour cells | [8] |
|  | KV11 is linked to exosomes by CP50 combined with CD63 | HUVEC | OIR mouse model | EXO_KV11_ suppresses vascular leakage in OIR model mice | [9] |
| **Biomimetic EVs** | PMA/Au-BSA@Ce6 nanocomposites are embedded into the exosomes by electroporation | urine from gastric cancer patients | human gastric carcinoma cell (MGC-803) tumor-bearing nude mice | Exo-PMA/Au-BSA@Ce6 nanovehicles could target tumor cells with deep penetration and superior retention performance in tumors | [10] |
|  | the boron nitride nanotubes loaded with doxorubicin and coated with cell membranes extracted from glioblastoma multiforme cells | glioblastoma multiforme cells | vitro multi-cellular and dynamic model | penetrate into glioma tissues and greatly promote cellular internalization and antiproliferation ability as well as extending circulation time | [11] |
|  | PMA/Fe-HSA@DOX, nano-sized Fe3O4 integrated with chemotherapeutics (DOX) via electrostatic adsorption of human serum albumin, are embedded into exosomes | prostate cancer patient's fasting urine | prostate cancer (DU145) tumor bearing mice | Enhance the cellular internalization, tumor targeting and deep penetration of biomimetic nanoparticles | [12] |
| **Genetic manipulation** | vectors encoding CTP-Lamp2b are introduced into HEK 293 cells to produce CTP-Lamp2b-modified exosomes | HEK 293 cells expressing CTP-Lamp2b | normal mice | expressing CTP-Lamp2b enhance exosome delivery to heart cells and the heart tissue | [13] |
|  | RNP‐enriched EVs were produced by co‐transfection of three plasmids into HEK293T cells | transfected HEK293T cells | del52hDMD/mdx mice | the RNP enriched EVs are efficient in genome editing and can achieve multiplex genome editing | [14] |
|  | fibroblasts were transduced with lentiviral vectors for overexpression of CD47 and/or GM‐CSF | transfected fibroblasts | cell line‐derived xenografts and patient‐derived tumor xenografts of metastatic peritoneal carcinoma | CD47 on exosome surfaces increases circulating time and enhances macrophage‐mediated tumor cell phagocytosis | [15] |
|  | si-c-Met were transfected with Lipofectamine 2000 and Opti-MEM | Transfected HEK293T cells | subcutaneous xenograft mouse model of Cisplatin-resistant gastric cancer | EXO-si-c-Met enhances the sensitivity of tumors to cisplatin in vivo | [16] |

c(RGDyK) peptide, cyclo(Arg-Gly-Asp-D-Tyr-Lys) peptide; SIRPα, signal regulatory protein alpha; HEK, human embryo kidney cells; NSG, NOD SCID gamma; PEG, polyethylene glycol; mTHPC, a fluorescent clinically approved antitumor photosensitizer; HUVEC, human umbilical vein endothelial cells; MSC, mesenchymal stem cell, MDCK, Mardin–Darby canine kidney; SKOV3, a human ovarian cancer cell; EVs, extracellular vesicles; PSA, prostate-specific antigen; PSMA, prostate-specific membrane antigen; GPI, glycosylphosphatidylinositol; EGFR, epidermal growth factor receptor; KV11, an anti-angiogenic peptide; CD63, a membrane protein of exosome; CP05, peptide bounding specifically to CD63; OIR, oxygen-induced retinopathy; EXO, exosome; DOX, doxorubicin; CTP, cardiac-targeting peptide; RNP, ribonucleoprotein; GM‐CSF, granulocyte‐macrophage colony‐stimulating factor; si-c-Met, siRNA against c-Met.

**Supplementary Table 2. Combinational antitumor strategies of exosomes as drug carriers.**

| **Combinational strategies** | | **Exosomes source** | **Loading methods** | **Cancer model** | **Outcome** | **References** |
| --- | --- | --- | --- | --- | --- | --- |
| **TNAs combined with chemotherapeutic agents** | anticancer drug 5-FU and miR-21 inhibitor oligonucleotide | THLG-293T or LG-293T cells | electroporation | subcutaneous xenografts of | combinational delivery effectively reverse drug resistance and significantly enhanced the cytotoxicity in 5-FU-resistant colon cancer cells | [17] |
|  | doxorubicin and cholesterol-modified miRNA 159 | THP-1 cells | incubation | xenograft-tumor model of triple-negative breast cancer | co-delivery of doxorubicin and Cho-miR159 induced synergistic therapeutic effects | [18] |
|  | oxaliplatin and PGM5 antisense RNA 1 | 293T cells | electroporation | xenograft mouse model of CRC | co-delivering PGM5- antisense RNA 1 and oxaliplatin reverses drug resistance in CRC | [19] |
| **Chemotherapy-photodynamic therapy** | ferroptosis inducer (Erastin) and photosensitizer (Rose Bengal) | HEK293T cells | sonication | xenograft C57BL/6 model of liver cancer | induce obvious ferroptosis in hepatocellular carcinoma with minimized toxicity | [20] |
|  | aggregation-induced emission luminogens and proton pump inhibitors | tumor-derived exosomes | electroporation and co-incubation are used to load PPI and TBP-2 respectively | gastric cancer subcutaneous model | show a high tumor growth inhibition rate, and promote tumor immunogenic death | [21] |
|  | doxorubicin and the photodynamic therapy agent 5-aminolevulinic acid | tumor-derived exosomes | incubation | HCT_116_ tumor-bearing mice models | the combination chemo-photodynamic therapy could significantly slow down tumor growth and prolong survival time of tumor-bearing mice | [22] |
| **Immunotherapy in combinational therapy** | HCC-targeting peptide (P47-P), α-fetoprotein epitope (AFP212-A2) and immunoadjuvant (HMGN1) | dendritic cell | exosomal anchor peptide CP05 | orthotopic HCC mice models | induce potent antitumor immune responses and provide long-term protective immune memory against tumor re-challenge | [23] |
|  | human neutrophil elastase (ELANE) and Hiltonol (TLR3 agonist) | breast cancer cells | electroporation | orthotopic TNBC mice models and patient-derived tumor organoids | enhance the immunogenicity of TNBC cells and indirectly activates tumor-infiltrating cDC1s in situ | [24] |
|  | radiation therapy and RGD-EVs loaded with siRNA against PD-L1 | ReNcell VM (ReN) cells | incubation | GBM-bearing mice | delivery of siRNA by RGD-EV reversed radiation-induced PD-L1 expression and activated antitumor immunity | [25] |
|  | galectin-9 siRNA and oxaliplatin | bone marrow mesenchymal stem cell | electroporation and incubation | orthotopic pancreatic ductal adenocarcinoma mice models | induce effective innate and adaptive immunity through enhanced ICD induction, improved DC maturation, reversed immunosuppression and increased infiltration of antitumoral cytotoxic T lymphocytes | [26] |
|  | GM‐CSF and docetaxel (DTX) | transfected fibroblasts | exosome–liposome hybrid nanoparticle | line‐derived xenografts and patient‐derived tumor xenografts | induce macrophage polarization and enhance macrophage‐mediated tumor cell phagocytosis | [15] |
|  | modifying exosomes through genetic display of both anti-human CD3 and anti-human HER2 antibodies | Transfected Expi293 cells | Genetic manipulation | human HER2 breast cancer xenograft mouse models | redirect and activate cytotoxic T cells toward attacking HER2-expressing breast cancer cell and exhibit highly potent and specific anti-tumor activity | [27] |
|  | oncolytic virus (OVs) and paclitaxel (PTX) | LL/2 mouse lung cancer cell | incubation | tumor-bearing NFKB-luc2 mice | improve tumor-selective delivery and enhance immunogenicity and infiltration of CD4^+^ and CD8+ T-cells | [28] |
| **Phytochemicals in combinational therapy** | superparamagnetic iron oxide nanoparticles (SPIONs) and curcumin (Cur) | RAW264.7 cells | electroporation | orthotopic glioma models | show a potent synergistic antitumor effect and significantly improve inhibition effect | [29] |

TNAs, therapeutic nucleic acids; 5-FU, 5-Fluorouracil; THLG, target-Her2-LAMP2-GFP; LG, lAMP2-GFP; THP-1, a kind of human monocytes; CRC, colorectal cancer; HEK, human embryo kidney cells; TBP-2, a type-I PDT AIEgen; HCC, hepatocellular carcinoma; HMGN1, high mobility group nucleosome-binding protein 1, an immunoadjuvant capable of promoting recruitment and activation of dendritic cells; TNBC, triple negative breast cancer; cDC1s, type one conventional dendritic cells; siRNA, small interfering RNA; PD-L1, programmed cell death ligand-1; GBM, glioblastoma; ReNcell VM (ReN) cells, a neural progenitor cell line derived from the ventral mesencephalon region of the human fetal brain; RGD-EV, EV modified with a brain-tumor-targeting cyclic RGDyK peptide; GM‐CSF, granulocyte-macrophage colony-stimulating factor; HER2, human epidermal growth factor receptor 2.

References

[1] T. Tian, H.X. Zhang, C.P. He, S. Fan, Y.L. Zhu, C. Qi, N.P. Huang, Z.D. Xiao, Z.H. Lu, B.A. Tannous, J. Gao, Surface functionalized exosomes as targeted drug delivery vehicles for cerebral ischemia therapy, Biomaterials 150 (2018) 137-149.

[2] W. Nie, G. Wu, J. Zhang, L.L. Huang, J. Ding, A. Jiang, Y. Zhang, Y. Liu, J. Li, K. Pu, H.Y. Xie, Responsive Exosome Nano-bioconjugates for Synergistic Cancer Therapy, Angew Chem Int Ed Engl 59(5) (2020) 2018-2022.

[3] L. Xu, F.N. Faruqu, R. Liam-Or, O. Abu Abed, D. Li, K. Venner, R.J. Errington, H. Summers, J.T. Wang, K.T. Al-Jamal, Design of experiment (DoE)-driven in vitro and in vivo uptake studies of exosomes for pancreatic cancer delivery enabled by copper-free click chemistry-based labelling, J Extracell Vesicles 9(1) (2020) 1779458.

[4] M. Piffoux, A.K.A. Silva, C. Wilhelm, F. Gazeau, D. Tareste, Modification of Extracellular Vesicles by Fusion with Liposomes for the Design of Personalized Biogenic Drug Delivery Systems, ACS Nano 12(7) (2018) 6830-6842.

[5] Y. Lin, J. Wu, W. Gu, Y. Huang, Z. Tong, L. Huang, J. Tan, Exosome-Liposome Hybrid Nanoparticles Deliver CRISPR/Cas9 System in MSCs, Adv Sci (Weinh) 5(4) (2018) 1700611.

[6] G. Ma, M. Severic, M. Barker, S. Pereira, A. Ruiz, C.C.L. Cheung, W.T. Al-Jamal, Dually targeted bioinspired nanovesicle delays advanced prostate cancer tumour growth in vivo, Acta Biomater 134 (2021) 559-575.

[7] M.J.W. Evers, S.I. van de Wakker, E.M. de Groot, O.G. de Jong, J.J.J. Gitz-Francois, C.S. Seinen, J.P.G. Sluijter, R.M. Schiffelers, P. Vader, Functional siRNA Delivery by Extracellular Vesicle-Liposome Hybrid Nanoparticles, Adv Healthc Mater 11(5) (2022) e2101202.

[8] S.A. Kooijmans, C.G. Aleza, S.R. Roffler, W.W. van Solinge, P. Vader, R.M. Schiffelers, Display of GPI-anchored anti-EGFR nanobodies on extracellular vesicles promotes tumour cell targeting, J Extracell Vesicles 5 (2016) 31053.

[9] X. Dong, Y. Lei, Z. Yu, T. Wang, Y. Liu, G. Han, X. Zhang, Y. Li, Y. Song, H. Xu, M. Du, H. Yin, X. Wang, H. Yan, Exosome-mediated delivery of an anti-angiogenic peptide inhibits pathological retinal angiogenesis, Theranostics 11(11) (2021) 5107-5126.

[10] S. Pan, L. Pei, A. Zhang, Y. Zhang, C. Zhang, M. Huang, Z. Huang, B. Liu, L. Wang, L. Ma, Q. Zhang, D. Cui, Passion fruit-like exosome-PMA/Au-BSA@Ce6 nanovehicles for real-time fluorescence imaging and enhanced targeted photodynamic therapy with deep penetration and superior retention behavior in tumor, Biomaterials 230 (2020) 119606.

[11] D. De Pasquale, A. Marino, C. Tapeinos, C. Pucci, S. Rocchiccioli, E. Michelucci, F. Finamore, L. McDonnell, A. Scarpellini, S. Lauciello, M. Prato, A. Larranaga, F. Drago, G. Ciofani, Homotypic targeting and drug delivery in glioblastoma cells through cell membrane-coated boron nitride nanotubes, Mater Des 192 (2020) 108742.

[12] S. Pan, Y. Zhang, M. Huang, Z. Deng, A. Zhang, L. Pei, L. Wang, W. Zhao, L. Ma, Q. Zhang, D. Cui, Urinary exosomes-based Engineered Nanovectors for Homologously Targeted Chemo-Chemodynamic Prostate Cancer Therapy via abrogating EGFR/AKT/NF-kB/IkB signaling, Biomaterials 275 (2021) 120946.

[13] H. Kim, N. Yun, D. Mun, J.Y. Kang, S.H. Lee, H. Park, H. Park, B. Joung, Cardiac-specific delivery by cardiac tissue-targeting peptide-expressing exosomes, Biochem Biophys Res Commun 499(4) (2018) 803-808.

[14] X. Yao, P. Lyu, K. Yoo, M.K. Yadav, R. Singh, A. Atala, B. Lu, Engineered extracellular vesicles as versatile ribonucleoprotein delivery vehicles for efficient and safe CRISPR genome editing, J Extracell Vesicles 10(5) (2021) e12076.

[15] Q. Lv, L. Cheng, Y. Lu, X. Zhang, Y. Wang, J. Deng, J. Zhou, B. Liu, J. Liu, Thermosensitive Exosome-Liposome Hybrid Nanoparticle-Mediated Chemoimmunotherapy for Improved Treatment of Metastatic Peritoneal Cancer, Adv Sci (Weinh) 7(18) (2020) 2000515.

[16] Q. Zhang, H. Zhang, T. Ning, D. Liu, T. Deng, R. Liu, M. Bai, K. Zhu, J. Li, Q. Fan, G. Ying, Y. Ba, Exosome-Delivered c-Met siRNA Could Reverse Chemoresistance to Cisplatin in Gastric Cancer, Int J Nanomedicine 15 (2020) 2323-2335.

[17] G. Liang, Y. Zhu, D.J. Ali, T. Tian, H. Xu, K. Si, B. Sun, B. Chen, Z. Xiao, Engineered exosomes for targeted co-delivery of miR-21 inhibitor and chemotherapeutics to reverse drug resistance in colon cancer, J Nanobiotechnology 18(1) (2020) 10.

[18] C. Gong, J. Tian, Z. Wang, Y. Gao, X. Wu, X. Ding, L. Qiang, G. Li, Z. Han, Y. Yuan, S. Gao, Functional exosome-mediated co-delivery of doxorubicin and hydrophobically modified microRNA 159 for triple-negative breast cancer therapy, J Nanobiotechnology 17(1) (2019) 93.

[19] B. Hui, C. Lu, J. Wang, Y. Xu, Y. Yang, H. Ji, X. Li, L. Xu, J. Wang, W. Tang, K. Wang, Y. Gu, Engineered exosomes for co-delivery of PGM5-AS1 and oxaliplatin to reverse drug resistance in colon cancer, J Cell Physiol 237(1) (2022) 911-933.

[20] J. Du, Z. Wan, C. Wang, F. Lu, M. Wei, D. Wang, Q. Hao, Designer exosomes for targeted and efficient ferroptosis induction in cancer via chemo-photodynamic therapy, Theranostics 11(17) (2021) 8185-8196.

[21] D. Zhu, T. Zhang, Y. Li, C. Huang, M. Suo, L. Xia, Y. Xu, G. Li, B.Z. Tang, Tumor-derived exosomes co-delivering aggregation-induced emission luminogens and proton pump inhibitors for tumor glutamine starvation therapy and enhanced type-I photodynamic therapy, Biomaterials 283 (2022) 121462.

[22] R. Qian, B. Jing, D. Jiang, Y. Gai, Z. Zhu, X. Huang, Y. Gao, X. Lan, R. An, Multi-antitumor therapy and synchronous imaging monitoring based on exosome, Eur J Nucl Med Mol Imaging 49(8) (2022) 2668-2681.

[23] B. Zuo, Y. Zhang, K. Zhao, L. Wu, H. Qi, R. Yang, X. Gao, M. Geng, Y. Wu, R. Jing, Q. Zhou, Y. Seow, H. Yin, Universal immunotherapeutic strategy for hepatocellular carcinoma with exosome vaccines that engage adaptive and innate immune responses, J Hematol Oncol 15(1) (2022) 46.

[24] L. Huang, Y. Rong, X. Tang, K. Yi, P. Qi, J. Hou, W. Liu, Y. He, X. Gao, C. Yuan, F. Wang, Engineered exosomes as an in situ DC-primed vaccine to boost antitumor immunity in breast cancer, Mol Cancer 21(1) (2022) 45.

[25] T. Tian, R. Liang, G. Erel-Akbaba, L. Saad, P.J. Obeid, J. Gao, E.A. Chiocca, R. Weissleder, B.A. Tannous, Immune Checkpoint Inhibition in GBM Primed with Radiation by Engineered Extracellular Vesicles, ACS Nano 16(2) (2022) 1940-1953.

[26] W. Zhou, Y. Zhou, X. Chen, T. Ning, H. Chen, Q. Guo, Y. Zhang, P. Liu, Y. Zhang, C. Li, Y. Chu, T. Sun, C. Jiang, Pancreatic cancer-targeting exosomes for enhancing immunotherapy and reprogramming tumor microenvironment, Biomaterials 268 (2021) 120546.

[27] X. Shi, Q. Cheng, T. Hou, M. Han, G. Smbatyan, J.E. Lang, A.L. Epstein, H.J. Lenz, Y. Zhang, Genetically Engineered Cell-Derived Nanoparticles for Targeted Breast Cancer Immunotherapy, Mol Ther 28(2) (2020) 536-547.

[28] M. Garofalo, A. Villa, N. Rizzi, L. Kuryk, B. Rinner, V. Cerullo, M. Yliperttula, V. Mazzaferro, P. Ciana, Extracellular vesicles enhance the targeted delivery of immunogenic oncolytic adenovirus and paclitaxel in immunocompetent mice, J Control Release 294 (2019) 165-175.

[29] G. Jia, Y. Han, Y. An, Y. Ding, C. He, X. Wang, Q. Tang, NRP-1 targeted and cargo-loaded exosomes facilitate simultaneous imaging and therapy of glioma in vitro and in vivo, Biomaterials 178 (2018) 302-316.
